# Indicator Regularized Non-Negative Matrix Factorization Method-Based Drug Repurposing for COVID-19

**DOI:** 10.3389/fimmu.2020.603615

**Published:** 2021-01-29

**Authors:** Xianfang Tang, Lijun Cai, Yajie Meng, JunLin Xu, Changcheng Lu, Jialiang Yang

**Affiliations:** ^1^ College of Information Science and Engineering, Hunan University, Changsha, China; ^2^ Department of Science, Geneis Beijing Co., Ltd., Beijing, China; ^3^ Academician Workstation, Changsha Medical University, Changsha, China

**Keywords:** COVID-19, drug repurposing, non-negative matrix factorization, semi-supervised learning, biological networks

## Abstract

A novel coronavirus, named COVID-19, has become one of the most prevalent and severe infectious diseases in human history. Currently, there are only very few vaccines and therapeutic drugs against COVID-19, and their efficacies are yet to be tested. Drug repurposing aims to explore new applications of approved drugs, which can significantly reduce time and cost compared with *de novo* drug discovery. In this study, we built a virus-drug dataset, which included 34 viruses, 210 drugs, and 437 confirmed related virus-drug pairs from existing literature. Besides, we developed an Indicator Regularized non-negative Matrix Factorization (IRNMF) method, which introduced the indicator matrix and Karush-Kuhn-Tucker condition into the non-negative matrix factorization algorithm. According to the 5-fold cross-validation on the virus-drug dataset, the performance of IRNMF was better than other methods, and its Area Under receiver operating characteristic Curve (AUC) value was 0.8127. Additionally, we analyzed the case on COVID-19 infection, and our results suggested that the IRNMF algorithm could prioritize unknown virus-drug associations.

## Introduction

Human coronaviruses (HCoVs) are a large family of enveloped, single-stranded, and positive-sense RNA viruses belonging to the subfamily orthocoronavirinae. They include α-coronavirus, β-coronavirus, γ-coronavirus, and δ-coronavirus (1). Commonly, the α-coronavirus and the β-coronavirus can only infect mammals, particularly humans (2). Besides, the spread of severe acute respiratory syndrome coronavirus (SARS-CoV) and Middle East respiratory syndrome coronavirus (MERS-CoV) in the last 2 decades has brought considerable risks to human life and caused substantial economic losses worldwide (3). At the end of 2019, another new pathogenic human coronavirus was named COVID-19, because of its long incubation period, which caused the infection to spread rapidly to all regions globally (4–7). According to reports through media, the total number of people infected with COVID-19 globally has reached 60 million and the number of deaths has exceeded the 1,500,000 marks. Many researches have focused on the formation, treatment, and vaccine of the COVID-19 (8–16). On October 22, 2020, Remdesivir has become the first officially approved COVID-19 treatment by the U.S Food and Drug Administration (FDA). However, high medical expenses prompt researchers to find alternative drugs with the same or similar efficacy as Remdesivir.

Drug repurposing aims to explore new treatment strategies to treat diseases based on the approved drugs that are outside the scope of the original medical indication. It has gained considerable attention that drug repurposing can significantly reduce time and labor costs than *de novo* drug development (17–19). For instance, the use of an anti-viral drug, like Ribavirin, could be used to cure infectious diseases such as Hepatitis C Virus (HCV), respiratory syncytial virus (RSV), and influenza B virus (IBV) (20). Lim et al. proposed Ribavirin could be the potential drugs for COVID-19 based on the clinical treatment (21). Muralidharan et al. discovered that a combination of three drugs was more effective than the use of any single drug alone against COVID-19 with the binding energy increasing nearly to 100% using sequential docking (22). This evidenced that existing drugs can be repositioned and used as a potential molecular target for the treatment of COVID-19. However, so far, only a few databases collate the potential related-drug, with the rapidly growing researches on the repositioning of drugs to treat COVID-19.

The success of traditional computational methods of drug repurposing relies on protein targets, sequences, and other biological data. However, the novel computational methods focus on the relationship between drug-targets using network approaches are dependent on the development of high-throughput technologies. According to the respective experimental validation, there is increasing evidence in different studies indicating that these novel computational methods are meaningful and useful. From the PPI network, Zhou et al. combined the virus-related proteins and the drugs that target the corresponding proteins to construct a network-based method aimed at identifying potential drugs against COVID-19 (23). Elsewhere, Gysi et al. developed multiple network strategies for specific drugs with potential efficacy against COVID-19 and utilized three rank aggregation methods (Borda’s count, the Dowdall method, and CRank) to assess the selected drugs and gave the final drug ranking (24). Consequently, Wang et al. employed hierarchical virtual screening methods to analyze the structure of COVID-19 and used the MM-PBSA-WSAS method that calculates the binding energy to obtain the prospective inhibitors of COVID-19 (25). Therefore, research to identify and develop more effective drugs to prevent and treat COVID-19 is urgently needed (26). Also, it is imperative to use novel computational methods to accelerate the corresponding research about the large-scale test to show the association of different drugs with COVID-19.

In our study, we built a novel virus-drug dataset and proposed the indicator regularized non-negative matrix factorization (IRNMF) method to predict the potential drugs for COVID-19, which is the first time to apply such algorithm in this area. The FDA approved anti-viral drugs against viruses like the α-coronavirus, β-coronavirus, influenza virus, and HIV was adopted to construct the virus-drug association dataset, which comprised of 34 viruses, 210 drugs, and 437 confirmed related virus-drug pairs. We used the virus-drug dataset to research the relationship between the existing drugs and COVID-19. Several studies have outlined that the association relationship between miRNA or lncRNA and biological processes or diseases could effectively be predicted by the non-negative matrix factorization (NMF) method (27, 28). Based on this framework, we proposed the IRNMF method to investigate the relationship between the existing drugs and COVID-19. Firstly, we calculated the drug and virus similarities extracted from the molecular drug information and the sequenced information on viruses. Secondly, we constructed a virus-drug interaction network based on the virus-drug association, the drug similarity matrix, and the virus similarity matrix. Thirdly, we introduced the indicator matrix into the non-negative matrix factorization algorithm to constrain the final association matrix, which could select the optimal related drug of COVID-19. Five-fold cross-validation (CV) was used to evaluate the IRNMF performance and its AUC value was 0.8127. Consequently, this IRNMF performance AUC value was compared to that of other methods: NMF (0.7968), IMC (0.7221), CMF (0.6470), and RLSMDA (0.7384). The obtained prediction results indicated that the proposed method owed the optimal performance in predicting the virus-drug association with the treatment of COVID-19. Moreover, we analyzed the cases of COVID-19 and MERS-CoV infections, and our results suggested that the IRNMF method could improve the efficiency of the unknown virus-drug associations towards the treatment of COVID-19.

## Methods

**Figure 1:**
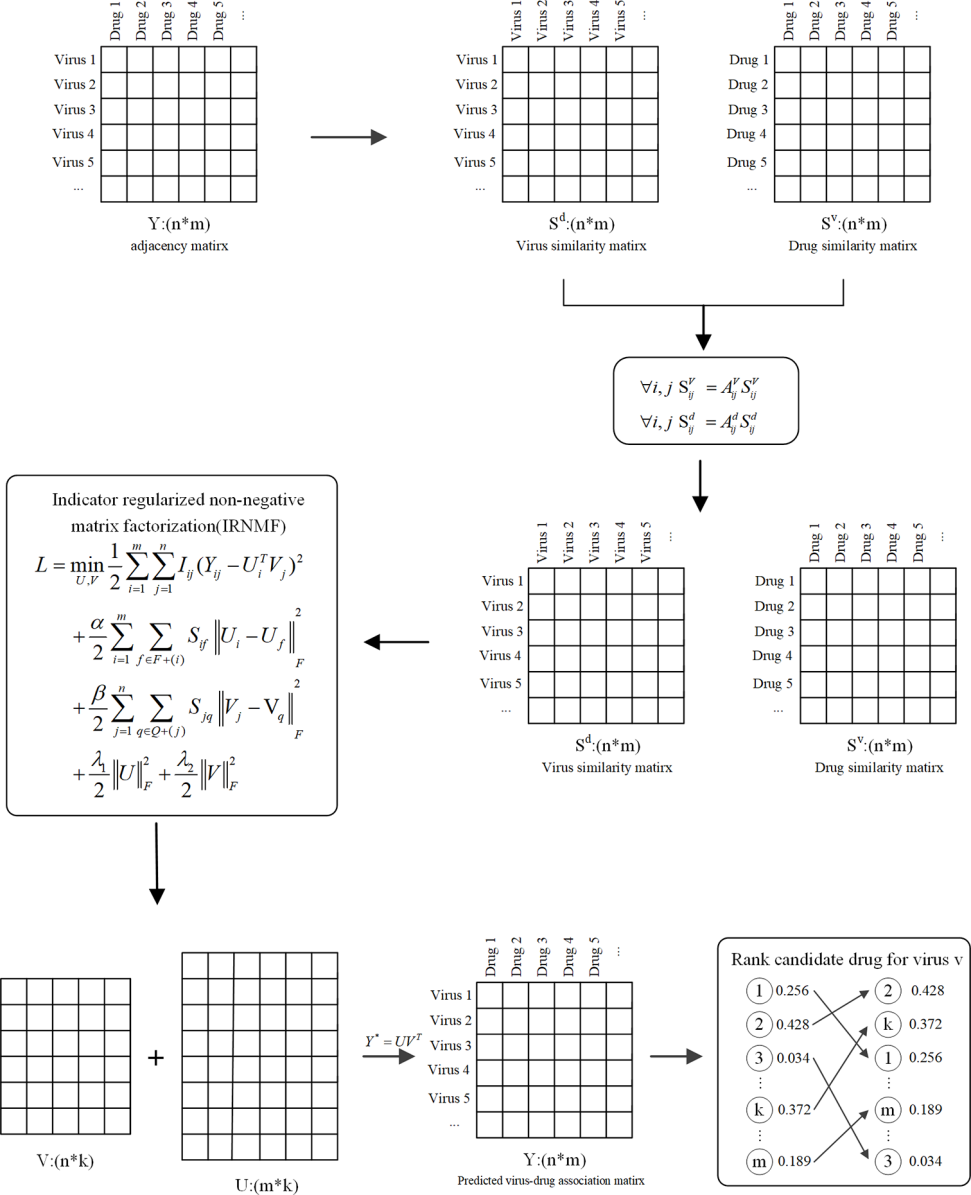
The framework of the IRNMF.

To detect the possible drugs against COVID-19, this study proposed a novel method called IRNMF, which includes three steps. First, we calculated the molecular similarity of the drugs and that of viruses. Next, the virus-drug interaction network based on the virus-drug association, drug, and virus similarity were constructed. Lastly, to reveal the potential drugs against COVID-19, we performed IRNMF. **Figure 1** shows the IRNMF method framework.

### Virus-Drug Datasets Collection

It is beneficial to provide novel strategies for the development of treatments against COVID-19 by conducting research on a variety of viruses and examining their corresponding drug targets. In terms of the virus collection, we preferred human infectious viruses, like coronavirus, RNA viruses, DNA viruses, and HIV. In the drug collection, original drugs against related viruses and broad-spectrum anti-viral drugs were used as therapeutic effects in treating viruses. About the above discussions, we constructed a virus-drug dataset, that included 34 viruses, 210 drugs, and 437 confirmed virus-drug pairs, as illustrated in **Table 1**.

**Table 1:** The virus-drug dataset.

Datasets	Virus	Drug	Virus-drug pairs
Numbers	34	210	437

### Virus Similarity Measure

Individual virus particles of the same strain of viruses have genetic sequences that are very similar, but not completely identical and this resemblance can be obtained by comparing different virus sequenced sequences. Multiple Alignment using Fast Fourier Transform (MAFFT) algorithm, which is a multiple sequence alignment program, is used for molecular biological studies. The MAFFT algorithm has been iteratively upgraded and has formed a complete system to help realize different biological information after performing a similarity analysis. This system has different methods like progressive, iterative refinement, and structural alignment for RNAs (29, 30). Here, we use the FFT-NS-1 algorithm to calculate the similarities of the viruses, which are appropriate for medium-scale alignments.

### Drug Similarity Measure

The existing drug similarity measurements were addressed based on the different overlap of viruses-related drugs. As shown in eq. (1), we adopted the Tanimoto coefficient (TC) to calculate the similarity between all drug pairs (31). This was about the molecular structure of drugs, which was downloaded from the DrugBank website.

(1) $$D(A,B)=\frac{S_{C}}{S_{A}+S_{B}-S_{C}}$$

S_A_/S_B_ belong to the value of drug A/B-related targets, genes and structures. S_C_ is the value of the Common parts between A and B. D (A, B) is the value of Tanimoto coefficient, which ranges from 0 to 1. The larger of the value, the more similar of the two drugs’ structure are. This calculation depended on Open Babel V2.3.1.

### Indicator Regularized Non-Negative Matrix Factorization

Several studies have focused on the matrix calculation found in biological analysis (32–34). The NMF method decomposes the origin matrix to a product of two non-negative matrices (35, 36). It could be used to convert the matrix Y of a defined virus-drug matrix *Y* ϵ *R^n^* ^×^ *^m^*, to two matrices: *U* ϵ *R^n^* ^×^ *^k^* and *V* ϵ *R^m^* ^×^ *^k^* (*k* << *min* (*n*,*m*))

(2) $$Y\approx UV^{T}$$

Based on the NMF method, we introduced the indicator matrix into the NMF to construct the IRNMF algorithm to repurpose virus-related drugs. This indicator matrix is used to ensure that both products of U and V are conversant with the original matrix. This intervention aims at effectively avoiding any noisy information, which only contains two values, either 0 or 1, whereby, 0 means no value at this position of the matrix, and 1 means there is a value at this position of the matrix.

(3) $$\begin{array}{l} L=\underset{U,V}{\min}\frac{1}{2}\sum_{i=1}^{m}\sum_{j=1}^{n}I_{ij}(Y_{ij}-U_{i}^{T}V_{j}{)}^{2}\\ \text{ +}\frac{\alpha }{2}Tr(U^{T}Q_{d}U)+\frac{\beta }{2}Tr(V^{T}Q_{v}V)\\ \text{ +}\frac{\lambda_{1}}{2}{||U||}_{F}^{2}+\frac{\lambda_{2}}{2}{||V||}_{F}^{2}\;s.t.\;U\geq 0,V\geq 0 \end{array}$$

Where I represent the indicator matrix, *α*, *β*, *λ*_1_, and *λ*_2_ are the regularization coefficients, *U_i_* and *V_j_* are the *i*th and *j*th rows of U and V respectively, and *S_if_* and *S_jq_* are the *i*th and *j*th rows of *S^d^* and *S^v^b* respectively, which belongs to drug similarity and virus similarity matrices respectively. The scaling factor ||‧||*_F_* is the Frobenius norm. Tr (.) is the trace value of the matrix; *Q_d_* = *D_d_* –*S^d^* and *Q_v_* = *D_v_* –*S^v^* are the Laplacian similarity matrix for *S^d^* and *S^v^*; where the values of *D_d_* and *D_v_* represents the diagonal matrices of *S^d^* and *S^v^* matrices, respectively (37).

(4) $$\begin{array}{l} L=\frac{I^{2}}{2}Tr(YY^{T})-I^{2}Tr(YU^{T}V)+\frac{I^{2}}{2}Tr(U^{T}VV^{T}U)\\ \text{ +}\frac{\alpha }{2}Tr(U^{T}Q_{d}U)+\frac{\beta }{2}Tr(V^{T}Q_{v}V)\\ \text{ +}\frac{\lambda_{1}}{2}{||U||}_{F}^{2}+\frac{\lambda_{2}}{2}{||V||}_{F}^{2}\\ \text{ +}Tr(\delta U^{T})+Tr(\epsilon V^{T}) \end{array}$$

According to the corresponding Lagrange function, *δ* and *ϵ* belongs to the matrix of the Lagrange factor, which are defined as *δ* =[*δ_ik_*] and *ϵ* = [*ϵ_jk_*]. Hence, it is easy to obtain the partial derivatives of the Eq. (4) using the values of U and V. We adopted the Karush-Kuhn-Tucker (KKT) condition to resolve the Lagrange optimization process. Defined as *δ_ik_U_ik_* = 0 and *ϵ_jk_ V_jk =_*0, and then we could get the regularized non-negative matrices U and V, which represented drug and virus matrices, respectively.

(5) $$\begin{array}{l} u_{ik}\frac{{\left(I^{2}VY+\alpha S_{d}V\right)}_{ik}}{{\left(I^{2}VV^{T}U+\alpha D_{d}V+\lambda_{1}U+\delta \right)}_{ik}}\to u_{ik}\\ v_{jk}\frac{{\left(I^{2}UY^{T}+\beta S_{v}V\right)}_{jk}}{\left(I^{2}UU^{T}V+\beta D_{v}V+\lambda_{2}V+\epsilon \right)}\to v_{jk} \end{array}$$

According to the Eq. (5), we could get the corresponding virus-drug association matrix by using the formula *Y* ^*^ = *U^T^V*, and then selecting the optimal virus-related drugs based on the matrix *Y* ^*^.

## Results and Discussion

### Performance Evaluation Metrics

To evaluate the performance of IRNMF and other methods with 5-fold CV (38). The virus-drug datasets were randomly divided into five subsets with equal sizes. In the experiment, four subsets were selected to train the model, respectively, and the last subset was used to evaluate the performance of the model. after the process was repeated for 5 times repeated, all the virus-drug of association scores have been estimated and sorted once.

Subsequently, we set a threshold *σ*. Here, if the association score was higher than *σ*, it was concluded that the prediction of the positive sample value was correct. However, if the association score was lower than *σ*, it meant that the prediction of the negative sample was correct. Consequently, this study adopted the receiver operating characteristic (ROC) curve to assess and compare these methods. The true positive rate (TPR) and the false positive rate (FPR) were compiled as follow:

(6) $$TPR=\frac{TP}{TP+FN},FPR=\frac{FP}{TN+FP}$$

TP and TN are the numbers of true positive and true negative samples that the method could predict whereas FP and FN are the numbers of false-positive and false-negative samples that the method could predict. Lastly, the area under the ROC curve (AUC) was used to calculate the performance of the different corresponding methods.

### Parameter of the IRNMF

In this work, the optimal parameters of the IRNMF method were determined using the following values: k is the value of low rank, which was set at 18. For *α*, *β*, *λ*_1_, and *λ*_2_, the values belonged to the regularization coefficients. To simplify this method, we assumed that *α* = *β* = 0.8 and *λ*_1_ = *λ*_2_ = 0.1. The value of iteration was set at 1500.

### Comparison With Other Methods

In this study, we adopted the IRNMF algorithm to select potential drug targets against COVID-19. Here, the database did not correlate the corresponding drug targets and COVID-19 and the predicted related drugs with other drugs and viruses. To confirm the performance of the IRNMF, we compared four algorithms based on the virus-drug network association prediction using different methods such as non-negative matrix factorization (NMF), Notably, all the above methods belonged to the semi-supervised procedures. Luo et al. adopted the NMF method and predicted small molecule-miRNA associations (28). Natarajan et al. proposed that the IMC method predicts gene-disease associations (39). The IMC method combines multiple types of features to learn latent factors, and it can be applied to the novel associations and it just needs the existing associations networks rather than the traditional matrix completions. Elsewhere, the CMF method was developed to predict the drug-disease association model (40). This method joins multiple matrices, which are factorized using the original interaction matrices, and represents the association of different classes, which helps to understand the potential characteristics of different relationships. Additionally, Chen et al. developed the RLSMDA algorithm to study the correlation between miRNAs and diseases (41). To obtain the likelihood of the relationship of miRNAs and diseases, this algorithm designed a continuous classification function using regularized least squares. Of note, before the experiments were performed in the above-stated comparison methods, the optimal parameter settings were adjusted accordingly.

**Figure 2:**
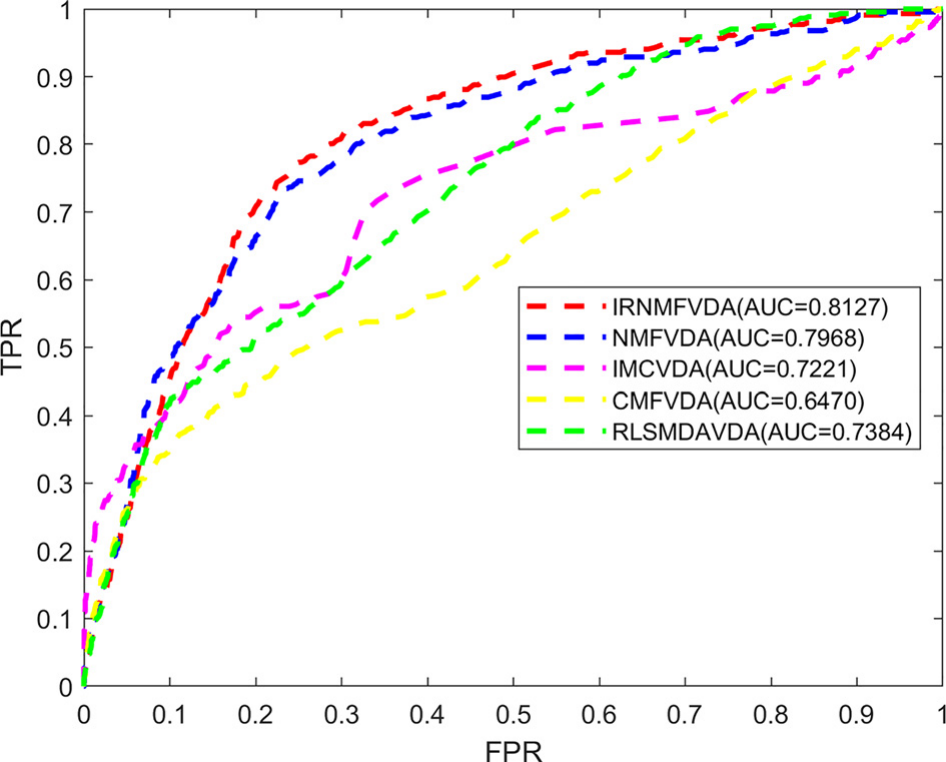
The comparison of methods. IRNMFVDA, NMF, IMCVDA, CMFVDA, RLSMDAVDA of ROC curve and AUCs value based on 5-fold CV.

Here, the proposed IRNMF method was compared with NMF, IMC, CMF, and RLSMDA methods through 5-fold CV. As illustrated in **Figure 2**, the IRNMF ROC curve value was above that of NMF, IMC, CMF, and RLSMDA in most of the experiments. The AUC values of IRNMF, NMF, IMC, CMF, and RLSMDA were 0.8127, 0.7968, 0.7221, 0.6470, and 0.7384, respectively. Notably, the AUC value of the IRNMF algorithm increased on average than the other four methods were 2%, 12.5%, 25.6%, and 10.1%, respectively. It demonstrated that the IRNMF mastered more knowledge about the similarity of viruses and that of drugs than the other methods, particularly, it showed a better prediction performance than NMF.

We also selected the most frequent potential drugs for COVID -19 through the IRNMF and the other 4 algorithms. As was the **Table 2** shown, Ribavirin, Nitazoxanide, Amantadine, N4-Hydroxycytidine, Chloroquine, and Mizoribine were predicted by all methods. Camostat, Niclosamide, Favipiravir, Zanamivir, Artesunate, Umifenovir, and Gemcitabine appeared 3–4 times. Particularly, the 13 predicted drugs belonged to the top16 potential drugs by the IRNMF method were the same as the most frequent potential drugs, and the other drugs top16 drugs selected by the IRNMF method have been predicted at least once by the four methods. The result showed that the top-ranked predictive drugs were more important than the lower-ranked predictive drugs and the valuable drugs were more possible to selected by the other methods. Therefore, we attest that the IRNMF algorithm is helpful for the prediction of drugs against COVID-19.

**Table 2:** The most frequent potential drugs for COVID -19 by the five methods.

IRNMF	Drugs	Statistics
1	**Ribavirin**	5
2	**Amantadine**	5
3	**Chloroquine**	5
4	**Nitazoxanide**	5
5	**N4-Hydroxycytidine**	5
6	**Camostat**	4
7	**Niclosamide**	4
8	**Berberine**	4
9	**Favipiravir**	3
10	**Zanamivir**	3
11	**Artemisinin**	3
12	**Umifenovir**	3
13	**Remdesivir**	3
14	Gemcitabine	3

The statistics based on the Top16 predicted drugs by the five methods. The bold drugs represent the predicted drugs by the IRNMF method.

### The Performance Influence of IRNMF Parameter

**Figure 3:**
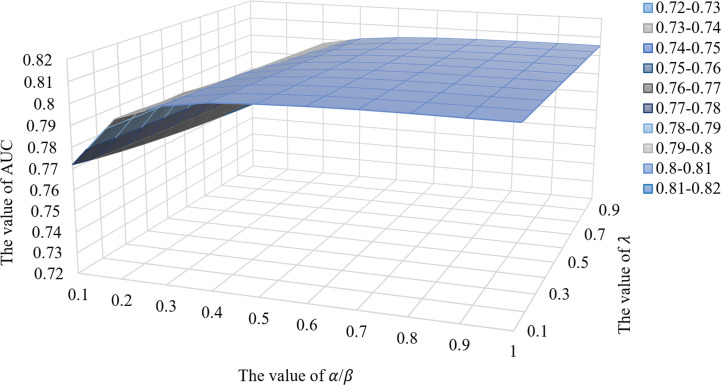
The performance influence of IRNMF parameter.

To assess the impact of IRNMF performance parameters, this study, assumed that the 4 parameters of the regularization coefficients were as follows; *α* = *β* and *λ*_1_ = *λ*_2_, and then the comparative experiments were performed by adjusting these two parameters. As shown in **Figure 3**, when the value of *α*/*β* was in the range of 0.1–0.4, the IRNMF performance could be improved if the value of *α*/*β* increased. However, when the value of *α*/*β* was fixed, the IRNMF performance was weakened when the value of *λ* increased. When the value of *α*/*β* was in the range of 0.5-1.0, the IRNMF performance was stable between 0.8 and 0.81, and the performance of IRNMF fluctuations was maintained at a small range. Our results showed that IRNMF had a stable generalization performance.

### IRNMF Identified of Potential Drug for COVID-19

**Figure 4:**
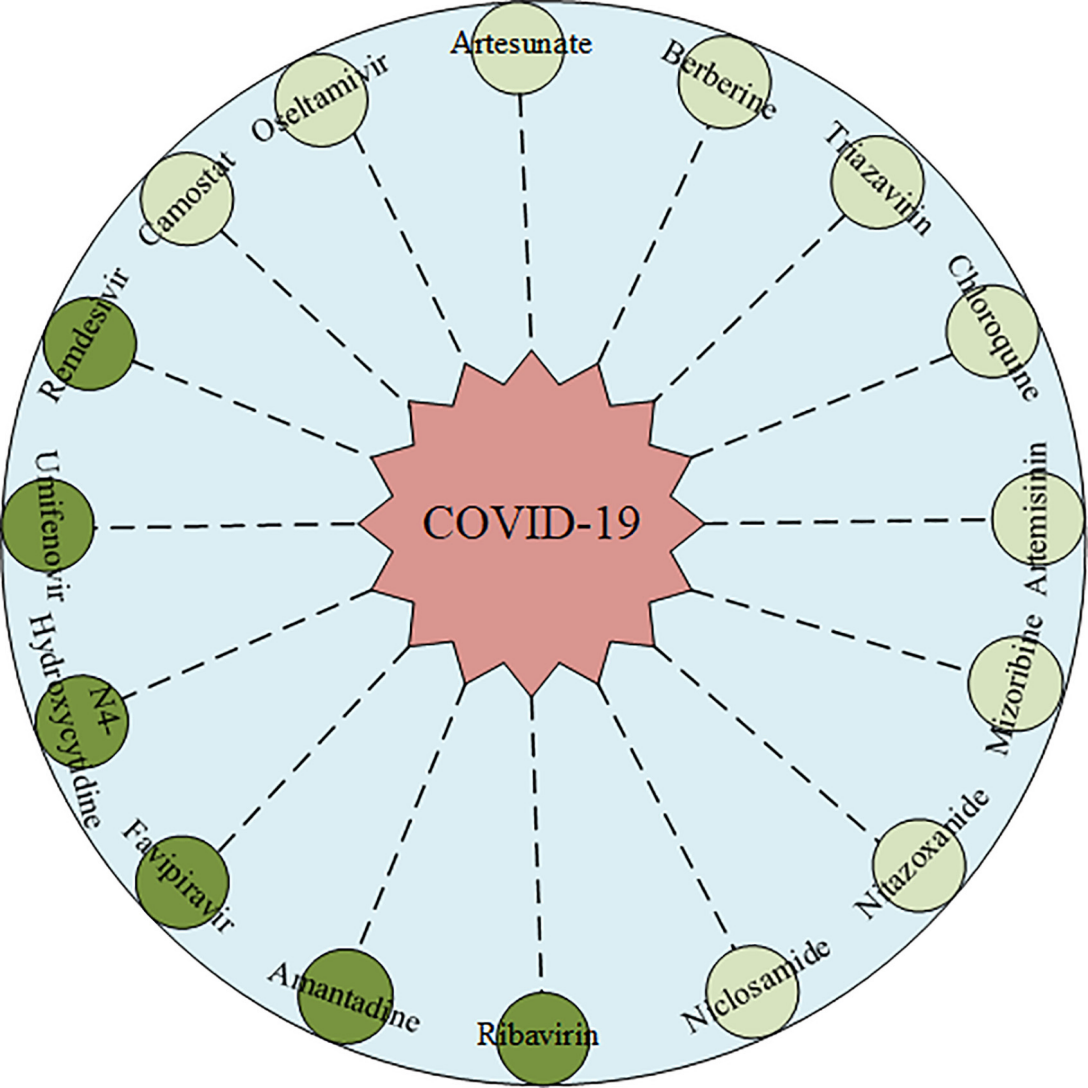
The predicted Top16 potential drugs by IRNMF. The dark color indicated the predicted drug has been validated. The light color indicated the predicted drug has not been validated.

In this study, we analyzed top16 predicted potential drugs against COVID-19. As illustrated in **Figure 4**. drugs such as Ribavirin, N4-Hydroxycytidine (NHC), Remdesivir, and Favipiravir, belong to similar nucleoside analogs and they can bind to the RNA dependent RNA polymerase (RdRps) enzyme which is crucial during the life cycle of RNA viruses. Ribavirin, an anti-viral drug is used to treat infections caused by influenza viruses and HCV. Hung et al. (42) recommended that the clinical trials, among the 127 patients during the randomized control trial, on the joint use of interferon beta-1b, lopinavir-ritonavir, and Ribavirin revealed that the joint use of these anti-viral drugs was safer and more effective than single medication used for the treatment of COVID-19. Elsewhere, the N4-Hydroxycytidine (NHC) has been demonstrated that it could treat infections caused by the influenza virus, Ebola, and SARS-CoV. Moreover, Sheahan et al. (43) noted that it could inhibit clinical isolates of COVID-19 infection through the replication of both Vero and Calu-3 cells. This study resulted in a dose-dependent reduction on the following; IC50 of 0.3 μM and CC50 >10μM in Vero cells, IC50 of 0.08 μM in virus titers, and IC50 of 0.09 μM in viral genomic RNA. Remdesivir which has been developed for the treatment of the Ebola virus, has been of interest after it showed the capability of metabolizing to Remdesivir triphosphate which competes for incorporation through RdRps and the fact that it interferes with the viral RNA replication of COVID-19 (13). Hillaker et al. conducted a study that showed that Remdesivir has a therapeutic effect on COVID-19 patients who stayed and received treatment in a mixed medical intensive care unit of the hospital (44). What’s more, Remdesivir has been recommended the first therapeutic drug for COVID-19 by the FDA. Favipiravir is an anti-viral drug, which is shown to treat the infections caused by the H1N1, H5N1, and Ebola viruses. For instance, Cai et al. confirmed that among the patients with COVID-19, before and after conducting an open-labeled controlled study on them, the administration of Favipiravir could significantly improve their state in terms of disease progression and viral clearance (45). Amantadine has been used for many years as one of the first-choice anti-viral drugs against influenza A viruses. Some studies have also proved that it could enter and block the viroporin channel to protect the cell cytoplasm from the released viral nuclei of coronaviruses like the SARS-CoV. Araújo et al. reported that if a patient takes 100mg of amantadine twice a day for 14 days his or her clinical status could improve (46). Umifenovir, is also widely used in the treatment of influenza viruses such as RSV, and SARS-CoV. A randomized clinical trial by Chen et al. confirmed that a 7-day recovery rate was observed after using Umifenovir. Here, out of 120 adult patients, 62 of them recovered from COVID-19 (47).

Oseltamivir, an orally administered neuraminidase (NA) inhibitor glycoprotein is majorly prescribed for persons with infections caused by influenza A and B viruses. Kongsaengdao et al. suggested that the combined use of oseltamivir, chloroquine, and Favipiravir could be beneficial in the treatment against COVID-19, and the clinical trial of this study showed an EC50 of 61.88 μM and CC50 > 400 μM (48). Zanamivir, a neuraminidase inhibitor that disrupts viral exit from infected cells and has known anti-viral activity that inhibits influenza viruses. Hall et al. found evidence that Zanamivir could be the 3CL protease proteinase inhibitor against COVID-19 infection using silicon docking models (49). Clinical trials on Nitazoxanide, which belong to the anti-viral prodrug, showed that this drug exhibits an *in vitro* activity against MERS-CoV and SARS-CoV infections, and therefore it could be used as a potential drug for the treatment of COVID-19 patients (50). Artemisinin is a potent anti-malarial drug. Sardar et al. has established that it is effective for the treatment of both COVID-19 and malaria co-infections (51). Hence, it is imperative to validate the role Artemisinin plays in the treatment of COVID-19. Artesunate- amodiaquine (AS-AQ), is also an antimalarial drug. Bae et al. suggested that it is highly effective against COVID-19 infection that develops in human lung epithelial cells (52). Berberine, an isoquinoline alkaloid, inhibits DNA synthesis and reverse transcriptase activity. In several studies, it has proved that it has an anti-viral activity against HSV, RSV, SARS-CoV, and HIV viruses (53). A certain study by Narkhede et al. purported that this alkaloid has favorable drug-like properties and it could be the potential drug against COVID-19 since it inhibits viral proteases characterized by the lowest binding energy (54). Ko et al. proved that Niclosamide, an anthelminthic drug could be the potential drug against COVD-19, since it blocks the endocytosis of COVID-19 and prevent its autophagy by inhibiting the S-Phase kinase-associated protein 2 (55). Camostat, is a synthetic serine protease inhibitor, which inhibits TMPRSS2 is also a potential drug against SARS-CoV and MERS-CoV infections. Uno et al. recommended that if a patient takes a daily dosage of 600mg it could effectively reduce the infections caused by COVID-19 (56). Therefore, there is a need for scientists to perform more experiments to validate this potential drug. Mizoribine is an immunosuppressive drug, and its research shows that it could inhibit the replication of SARS-CoV and MERS-CoV infections, hence it could also be a potential drug against COVID-19.

In addition, Chloroquine, an antimalarial drug, is used in the treatment of several other diseases. Gao et al. found that chloroquine could interfere with the general endocytic trafficking to inhibit both the replication and infection caused by COVID-19 (57). However, Mehra et al. proposed that chloroquine could not help patients’ recover when used alone or with a macrolide (58). Based on these, we don’t recommend this drug as a clinical treatment option.

## Conclusion

In conclusion, COVID-19 is becoming one of the most prevalent and infectious diseases in human history. Existing drugs can be repositioned and used as a potential molecular target for the treatment of COVID-19. However, so far, only a few databases collate the potential related-drug to treat COVID-19. In this study, we constructed the virus-drug dataset, which included 34 viruses and 210 drugs, which were obtained from 437 confirmed cases on the related pairs about the virus and drug. Besides, we developed the indicator regularized non-negative matrix factorization (IRNMF) algorithm to predict the potential drug against COVID-19. In this IRNMF model, the known virus-drug associations, the virus similarity networks, and the drug similarity network were merged to calculate the prediction score of each virus-drug pair. According to the 5-cross VDA, the AUC value of IRNMF is 0.8127. Comparing this value with that of NMF (0.7968), IMC (0.7221), CMF (0.6470), and RSLMDA (0.7384), this proposed method achieved better performance. The results suggest that the IRNMF algorithm can deduce the unknown virus-drug associations.

In addition, IRNMF is restricted with the scale of the virus-drug dataset, the predicted potential drugs might not be totally accurate. Therefore, according to the corresponding drug and virus database, our future work is to enlarge the virus-drug dataset of COVID-19.

## Data Availability Statement

The datasets presented in this study can be found in online repositories. The names of the repository/repositories and accession number(s) can be found below: https://github.com/dukebai/IRNMF.

## Author Contributions

XT: Conceptualization, methodology, software, writing—original draft, visualization. JX, JY, LC: Conceptualization, methodology, visualization, supervision, project administration, funding acquisition. YM, CL: data curation, formal analysis, writing—review and editing. All authors contributed to the article and approved the submitted version.

## Funding

This study was partially supported by the Hunan Provincial Innovation Foundation for Postgraduate under grant no. CX20200434, the Natural Science Foundation of Hunan Province (Grant No. 2018JJ2461), and the project to introduce intelligence from oversea experts to the Changsha City (No. 2089901).

## Conflict of Interest

JY was employed by Geneis Beijing Co., Ltd.

The remaining authors declare that the research was conducted in the absence of any commercial or financial relationships that could be construed as a potential conflict of interest.
